# L'impact du vieillissement démographique sur la consommation médicale: assurance maladie obligatoire au Maroc

**DOI:** 10.11604/pamj.2020.35.93.20716

**Published:** 2020-03-27

**Authors:** Imane Sninate, Ahmed Bennana

**Affiliations:** 1 Université Mohammed V-Souissi, Faculté de Médecine et de Pharmacie de Rabat, Centre d'Etudes Doctorales en Sciences de la vie et de la Santé, Rabat, Maroc

**Keywords:** Assurance maladie obligatoire de base, affections de longue durée, vieillissement démographique, dépense médicale moyenne par personne couverte, Standard compulsory health insurance, diseases of long duration, demographic ageing, average medical expenditure per covered person

## Abstract

**Introduction:**

les régimes d'assurance maladie obligatoire des salariés des secteurs public et privé connaissent, d'une part, une croissance continue des dépenses médicales, et d'autre part, une augmentation de la part de la population couverte ayant plus de 60 ans. L'objectif de cet article est d'analyser l'impact du vieillissement sur la dépense moyenne par personne bénéficiant de la couverture médicale.

**Méthodes:**

nous avons effectué le test de Kruskal-Wallis afin de tester si il y a une différence significative entre les populations - de différents groupes d'âge - en matière de consommation médicale selon que la personne est touchée ou non par une affection de longue durée. Ont été effectuées également des analyses d'autres indicateurs à savoir la part de la population souffrant d'une affection de longue durée et recourant aux soins, la dépense moyenne par personne touchée par une affection de longue durée et la dépense moyenne par personne couverte non touchée par une affection de longue durée aussi bien pour les personnes ayant moins de 60 ans que celles ayant plus de 60 ans.

**Résultats:**

le test de Kruskal-Wallis a montré que l'âge est une variable explicative de la dépense moyenne par personne couverte. L'examen de l'évolution des principales composantes de la dépense moyenne a permis de mettre en évidence d'autres facteurs.

**Conclusion:**

le vieillissement démographique contribuerait à la hausse de la consommation médicale et il existe d'autres facteurs dont l'impact sur l'augmentation des dépenses n'est pas moins important.

## Abstract (en)

**Introduction:**

compulsory health insurance schemes for all employees in public and private sector are causing, on the one hand, continual health care spending growth and, on the other hand, are experiencing an increase in the number of covered population aged over 60 years. The purpose of this study was to analyze the impact of ageing on the estimated average expenditure per covered person with health insurance.

**Methods:**

we conducted Kruskal-Wallis test to assess whether there was any significant difference between populations of different age on medical consumption, depending on whether the person was affected or not by a diseases of long duration. Other indicators were also analyzed, namely the proportion of the population suffering from a disease of long duration demanding health care, the average expenditure per person affected by a diseases of long duration and the average expenditure per covered person not affected by a disease of long duration for both people under 60 years and those over 60 years.

**Results:**

Kruskal-Wallis test showed that age is an explanatory variable for the average expenditure per covered person. The examination of the evolution of the main components of average expenditure allowed to highlight other factors.

**Conclusion:**

population ageing contributes to the increase of medical consumption but there are other factors which have a significant effect on the increase in expenditure.

## Introduction

La hausse continue des dépenses en soins de santé fait ressurgir des inquiétudes quant à la viabilité des systèmes d'assurance maladie. Le vieillissement démographique y est souvent associé car l'avancement en âge entraîne souvent la détérioration de l'état de santé et par conséquent l'augmentation du recours aux soins. Toutefois, de nombreux travaux dans la littérature récente considèrent le vieillissement comme un facteur secondaire. Ainsi, Zweifel *et al*, 1999 [1] et Brockmann (2000) [2] ont démontré que ce n'est pas l'âge mais la proximité du décès qui est déterminante dans les coûts des soins de santé. José Martin *et al* (2013) [3], dans leur revue de la littérature sur les déterminants de la consommation médicale portant sur 20 articles, avancent « qu'il n'existe pas de preuve empirique solide appuyant le fait que le vieillissement de la population est l'un des principaux déterminants des dépenses de santé». Les travaux de Cutler (1998) [4], Fuchs (1999) [5], Breyer et Felder (2006) [6] ont mis en avant le rôle important joué par l'expansion de la technologie dans l'explication de la hausse des dépenses.

Au Maroc, les régimes d'assurance maladie obligatoire (AMO) des salariés des secteurs public et privé gérés respectivement par la Caisse Nationale des Organismes de Prévoyance Sociale (CNOPS) et la Caisse Nationale de Sécurité Sociale (CNSS) connaissent une augmentation continue des dépenses médicales et font face à un poids important des dépenses liées aux affections de longue durée (ALD) qui correspondent aux maladies chroniques figurant sur la liste des quarante et une affections de longue durée faisant l'objet de l'arrêté du ministère de la santé n°2518-05 (5 Septembre 2005) [7]. Ainsi, la dépense moyenne par personne couverte a connu une évolution annuelle moyenne de 6% dans le secteur public et de 8,5% dans le secteur privé entre 2012 et 2015. En outre, les ALD touchent 2,8% de la population couverte et s'accaparent 48,2% des dépenses totales pour les deux régimes d'assurance maladie.

La hausse de la dépense moyenne par personne couverte pourrait être imputée à l'évolution démographique et épidémiologique de la population, le changement du comportement des assurés en termes de consommation médicale et des médecins en termes de prescription. Plus particulièrement, le vieillissement démographique pourrait également avoir une contribution à la hausse des dépenses de santé eu égard aux changements qu'a connus la structure démographique des populations couvertes et à l'écart entre les niveaux de consommation des personnes âgées et des personnes ayant moins de 60 ans. Ainsi, la part de la population ayant plus de 60 ans à la CNSS a été de 7,7% [8], 7,8% [9], 8,3% [10] et 8,4% [11] respectivement en 2012, 2013, 2014 et 2015. En ce qui concerne la CNOPS, la part de la population âgée de plus de 60 ans par rapport à la population totale a représenté 15,2% [8],15,5% [9], 16,0% [10] et 16,4% [11] respectivement en 2012, 2013, 2014 et 2015. De plus, la dépense moyenne par personne couverte ayant plus de 60 ans représente 6,4 fois la dépense moyenne par personne ayant moins de 60 ans à la CNSS et 2,7 fois à la CNOPS au titre de 2015. L'objectif du présent travail est d'approcher la place du vieillissement démographique dans l'évolution des dépenses médicales.

## Méthodes

Il s'agit d'une étude statistique visant, dans un premier temps, l'analyse de l'influence de la variable âge sur la dépense moyenne par personne couverte. Et ce, via l'application du test de Krushall-Wallis comme alternative non paramétrique à l'analyse de la variance-ANOVA-étant donné que l'hypothèse de la normalité de la distribution n'est pas vérifiée afin de tester s'il y a une différence significative entre les populations de différents groupes d'âge en matière de consommation médicale. Puis, vu l'impossibilité d'appliquer une ANOVA à deux facteurs avec interaction où la variable dépendante est la dépense moyenne par personne couverte et les variables explicatives sont l'ALD et la tranche d'âge, nous avons refait le même test après distinction entre la population touchée par une ALD de celle qui ne l'est pas. Dans un second temps, ont été présentés également des indicateurs permettant la compréhension de la dynamique des dépenses par tranches d'âge et l'évolution du profil de consommation des personnes ayant plus de 60 ans et ceux ayant moins de 60 ans. Les données sur lesquelles s'est basée la présente étude correspondent aux données transversales de la population couverte, la population atteinte d'au moins une ALD et la consommation médicale pour les régimes AMO gérés par la CNSS et la CNOPS ressorties des rapports annuels globaux de l’AMO produits par l'Agence Nationale de l'Assurance Maladie au titre de la période 2009- 2015 [11].

## Résultats

Dans le cas où le test de Krushall-Wallis mène au rejet de l'hypothèse de l'égalité des moyennes, on mène un test de Mann-Whitney pour analyser les groupes d'âge deux à deux afin d'identifier les tranches d'âges responsables du rejet de l'hypothèse qui stipule qu'il n'y a pas de différence significative entre les différentes tranches d'âge. Les résultats de ces deux tests se présentent comme suit (Tableau 1): pour la population couverte auprès de la CNOPS, il s'avère qu'il y a une différence significative entre quatre tranches d'âge en matière de dépense médicale moyenne. Plus particulièrement, la dépense moyenne par personne couverte diffère significativement entre 0-15 ans et 30-45 ans (p = 0,006 < 0,05), 0-15 ans et 45-60 ans (p = 0,000 < 0,05), 0-15 ans et plus de 60 ans (p = 0,001 < 0,05). En ce qui concerne la population touchée par au moins une ALD, il n'y a pas de différence significative entre la dépense moyenne par personne couverte pour les différentes tranches d'âge.

**Tableau 1:** Résultats du test de Krushall-Wallis et de Mann-Whitney

Tranches d'âge	CNOPS			CNSS		
	ALD=“Oui”	ALD=“Non”	Total	ALD=“Oui”	ALD=“Non”	Total
**Kruskal-Wallis Test**	**0,388**	**0,000**	**0,003**	**0,000**	**0,000**	**0,000**
Mann-Whitnney Test						
0-15 et 15-30		0,470	0,064	0,063	0,305	0,377
0-15 et 30-45		0,003	0,006	0,216	0,002	0,001
0-15 et 45-60		0,000	0,000	0,074	0,000	0,000
0-15 et plus de 60 ans		0,001	0,001	0,143	0,000	0,000
15-30 et 30-45		0,052	0,560	0,363	0,000	0,039
15-30 et 45-60		0,000	0,149	0,543	0,000	0,023
15-30 et plus de 60 ans		0,005	0,244	0,429	0,000	0,002
30-45 et 45-60		0,000	0,173	0,815	0,670	0,761
30-45 et plus de 60 ans		0,043	0,222	0,959	0,000	0,064
45-60 et plus de 60 ans		0,757	0,858	0,621	0,000	0,134

Alors que pour la population non touchée par une ALD, les tranches d'âges qui ne différent pas significativement en termes de consommation médicale moyenne sont 0-15 et 15-30 ans (p = 0,470 > 0,05), (15-30ans) et (30-45 ans) (p = 0,052 > 0,05) et (45-60 ans) et (plus de 60 ans) (p = 0,757 > 0,05). Pour la population totale couverte auprès de la CNSS, le test de Krushall-Wallis montre que l'âge est une variable qui influence significativement la dépense moyenne par personne couverte (p = 0,000 < 0,05). Les comparaisons multiples entre les tranches d'âge deux à deux montrent qu'il n'y a pas de différence significative entre (0-15) et (15-30) (p=0,377> 0,05), (30-45) et 45-60 (p = 0,761 > 0,05), 30-45 et plus de 60 ans (p = 0,064 > 0,05), 45 - 60 et plus de 60 ans (p = 0,134 > 0,05). Pour la population qui n'est pas touchée par au moins une ALD, il y a une différence significative entre toutes les tranches d'âge à part les couples 0 - 15 et 15-30 (p = 0,305 > 0,05) ainsi que 30-45 et 45-60 (p = 0,670 > 0,05).

En ce qui concerne la population qui souffre d'au moins une ALD, le test de Krushall-Wallis a montré qu'il y a une différence significative entre la dépense moyenne par personne couverte des différentes tranches d'âge alors que les résultats du test de Mann-Whitney montrent qu'il n'y a pas de différence significative entre les différentes tranches d'âge. Le Tableau 2 ci-dessous présente l'évolution de la dépense moyenne par personne couverte selon la tranche d'âge. Le ratio de la dépense moyenne par personne touchée par une ALD comparativement à la dépense moyenne d'une personne Non ALD est de 46,4 et 17,4 respectivement à la CNSS et la CNOPS. Pour la population qui n'est pas touchée par une ALD à la CNOPS, la dépense moyenne par personne couverte varie entre 283 Dhs et 383 Dhs pour la population âgée entre 1 et 15 ans. Entre la tranche 20-25 et 25-30, la dépense moyenne par personne couverte a connu une augmentation de 345% passant de 253 Dhs à 1126 Dhs.

**Tableau 2:** La dépense moyenne par personne couverte selon la tranche d'âge et selon que la personne est touchée par une ALD ou Non (En Dirhams)

Tranches d'âge	CNOPS		CNSS			
	ALD=“Non”	ALD=“Oui”	Total	ALD=“Non”	ALD=“Oui”	Total
0-1	726	25 533	740	188	27 523	196
1-5	383	17 622	430	107	8 271	117
5-10	283	9 389	333	69	4 571	79
10-15	339	12 494	419	57	6 419	79
15-20	345	18 057	471	71	5 797	93
20-25	253	12 488	342	131	11 545	163
25-30	1 126	16 172	1 313	208	17 474	267
30-35	1 182	19 291	1 455	233	15 280	335
35-40	1 119	17 879	1 455	232	14 480	397
40-45	1 030	16 746	1 478	225	11 681	441
45-50	1 122	15 886	1 809	243	11 939	630
50-55	1 248	14 397	2 234	287	10 330	825
55-60	1 377	13 847	2 780	350	9 387	1124
60-65	1 502	14 065	3 388	533	8 965	1639
65-70	1 757	14 108	4 118	623	8 085	2041
plus de 70 ans	1 031	13 098	2 430	689	7 345	2091
**Total**	**817**	**14 260**	**1 433**	**200**	**9276**	**437**

Pour les tranches d'âge 30-45, 45-60 et plus de 60 ans, la dépense moyenne par personne couverte pondérée est respectivement de 1107 Dhs, 1249 Dhs et 1297 Dhs. A part la tranche 5-10 où la dépense moyenne par personne ALD est de 9389 Dhs et la tranche d'âge (0-1) où elle est de 25533 Dhs, la dépense moyenne par personne ALD à la CNOPS varie entre 12488 et 19 291Dhs. En ce qui concerne la CNSS, la dépense moyenne-pondérée par les effectifs-par personne couverte non touchée par une ALD est passée de 92 Dhs pour la tranche (0-15) à 141 Dhs pour la tranche (15-30). Puis elle atteint 230 Dhs et 283 Dhs respectivement pour les tranches 30-45 et 45-60. Quant à la tranche (plus de 60 ans), la dépense moyenne est de 615 Dhs. A l'instar de la CNOPS, la dépense moyenne par personne ALD la moins élevée est enregistrée au niveau de la tranche (5-10) où elle est de 4571 Dhs et la plus élevée au niveau de la tranche (0-1) où elle est de 27 523 Dhs. A part les tranches (0-1) et (5-10), la dépense moyenne par personne ALD varie de 5 797 Dhs à 17 474 Dhs.

Contrairement à la dépense moyenne par personne non ALD qui connaît une augmentation selon l'âge, la dépense moyenne par personne ALD connaît des fluctuations. Ceci nous amène à analyser la répartition des affections de longue durée dont est touchée la population atteinte par une ALD au sein de chaque tranche d'âge pour expliquer la différence du coût moyen par tranche d'âge. Pour la CNSS, les premières pathologies les plus prépondérantes, en termes de dépenses, diffèrent d'une tranche d'âge à l'autre. Ainsi, la cardiopathie congénitale représente 76,2% des dépenses ALD afférentes à la tranche 0-1. En ce qui concerne la tranche (1-20), l'insuffisance rénale chronique terminale (IRCT) (28,4%), affections malignes du tissu lymphatique (17%), épilepsie grave (8,8%), troubles héréditaires de l'hémostase (6,6%), tumeurs malignes (TM) (6%) et diabète (5,2%) consomment 72,2% des dépenses ALD liées à cette tranche. Quant à la tranche 20-40, l'IRCT (51%) et les TM (17,2%) s'accaparent 68,2% des dépenses concernant cette tranche.

Ces deux dernières pathologies sont les plus importantes en termes de dépenses également pour la tranche 40 - 60 ans: IRCT (41,1%) et TM (27,4%). Quant à la population ayant plus de 60 ans, l'IRCT (34,9%), l'hypertension artérielle (17,3%) et les TM (16,1%) représentent 68,4% des dépenses ALD de cette tranche. Quant à la CNOPS, les pathologies les plus fréquentes chez les nouveau-nés sont la cardiopathie congénitale (48,7%) et l'asthme sévère (18,3%). En ce qui concerne la population ayant entre 1 et 20 ans, cinq pathologies s'accaparent 51,9% des dépenses ALD afférentes à cette tranche d'âge: valvulopathies rhumatismales (19,1%), troubles héréditaires de l'hémostase (12,6%), le diabète ( 9,1%) et l'IRCT (6,3%). Pour ce qui est de la population âgée de 20 à 40 ans, quatre pathologies représentent 61,6% des dépenses totales liées aux ALD à savoir l'IRCT (26,4%), les TM (16%), la sclérose en plaques (15,3%) et les affections malignes du tissu lymphatique ou hématopoïétique (3,9%).

**Figure 1:**
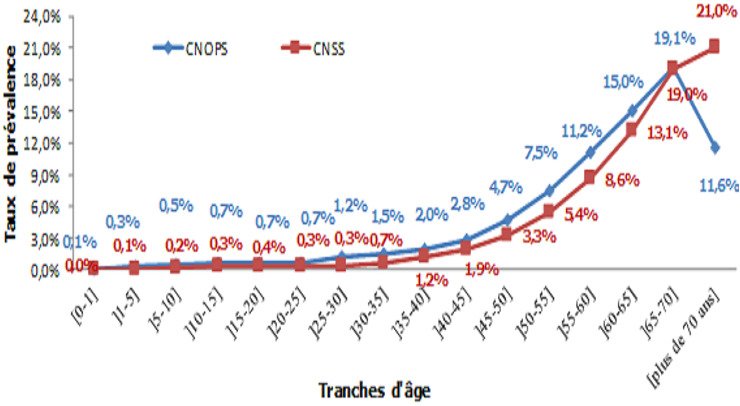
Taux de prévalence en affection longue durée

Quant à la population âgée entre 40 et 60 ans, elle a une consommation de 60,9% relativement à cinq pathologies à savoir les TM (26,4%), l'IRCT (16%), le diabète (10,5%), les affections malignes du tissu lymphatique ou hématopoïétique( 3,9%) et l'HTA (3,8%). Pour les personnes ayant plus de 60 ans, les cinq premières pathologies-en termes de dépenses liées aux ALD sont: l'IRCT (16,3%), TM (14,4%), l'HTA (9,3%), diabète (8,1%), les Affections malignes du tissu lymphatique (2,8%) qui totalisent 50,8% des dépenses totales liées aux ALD. Les dépenses totales liées aux ALD sont déterminées par le produit de trois paramètres à savoir la population couverte, le taux de prévalence en ALD et la dépense moyenne par une personne touchée par une ALD. Ainsi, il faut analyser la tendance de l'évolution de la prévalence en ALD par tranche d'âge (Figure 1). Même si le coût moyen d'une ALD dans les tranches d'âge jeunes est relativement élevé, la part des personnes touchées par une ALD dans ces tranches d'âge demeure moins importante que chez les personnes âgées.

Le taux de prévalence en ALD est moins de 1% pour les âges inférieurs à 25 ans et les âges inférieurs à 35 ans respectivement à la CNOPS et à la CNSS. Puis, il connaît une augmentation continue passant de 2% à 19,1% entre 35 et 70 ans à la CNOPS et de 2% à 19% à la CNSS entre 45 et 70 ans. Après avoir mis en évidence l'impact de l'âge, il convient de présenter l'évolution des principaux déterminants de la dépense médicale-qui ressortent de la formule ci-dessous-dans la limite des données disponibles à savoir le taux de recours aux soins (ou de sinistralité) des personnes ALD, la dépense moyenne par personne ALD ayant recouru aux soins (sinistrée) et la dépense moyenne par personne Non ALD.

Dépenses médicales = (population couverte* (part de la population qui en ALD et qui recourt aux soins * dépense moyenne par personne sinistrée ALD) + (population couverte *(1 - taux de prévalence en ALD) * dépense moyenne par personne couverte Non ALD).

**Figure 2:**
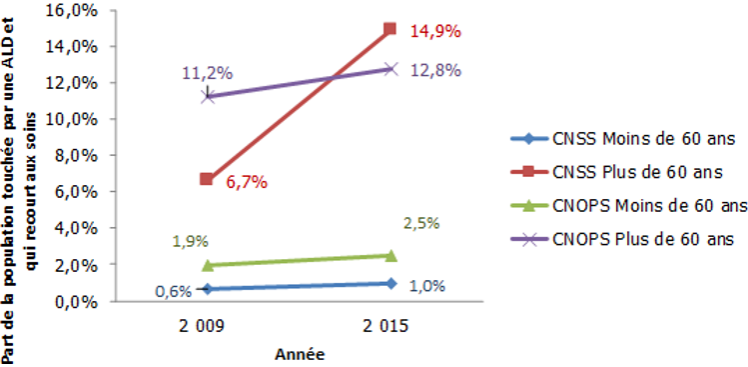
Évolution de la part de la population atteinte d'au moins une affection longue durée et qui recourt effectivement aux soins

**Figure 3:**
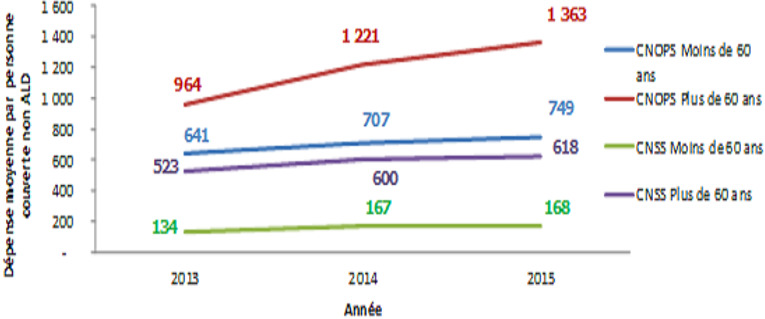
Évolution de la dépense moyenne par personne couverte non touchée par une longue durée

La Figure 2 montre que la part de la population ALD sinistrée est plus élevée chez les personnes âgées et a connu une évolution annuelle moyenne de 2,2% pour la CNOPS et de 14,4% pour la CNSS entre 2009 et 2015 pour la population ayant plus de 60 ans. Ce taux d'évolution a été de 7,1% pour les moins de 60 ans à la CNSS et de 4,2% à la CNOPS. La dépense moyenne par personne ALD ayant plus de 60 ans recourant effectivement aux soins se situe à des niveaux élevés: elle a varié entre 13798 Dhs et 16594 Dhs durant la période 2009-2015 pour la CNOPS et entre 6598 Dhs et 10050 Dhs durant la même période pour la CNSS. Pour les personnes ayant moins de 60 ans, cette dépense a varié entre 14102 et 18033 entre 2009 et 2015 à la CNOPS et de 10066 et 14394 à la CNSS durant la même période. En ce qui concerne la dépense moyenne par personne couverte non touchée par une ALD, la Figure 3 montre qu'elle a connu pour les personnes ayant plus de 60 ans une augmentation annuelle moyenne de 18,9% à la CNOPS et de 8,7% à la CNSS entre 2013 et 2015. Alors que pour les personnes ayant moins de 60 ans, cette dépense a connu une évolution annuelle moyenne de 8,1% à la CNOPS et de 12,1% à la CNSS durant la même période.

## Discussion

La présente analyse a montré que le vieillissement démographique contribuerait à la hausse de la consommation médicale et a mis en évidence également la présence d'autres facteurs dont l'impact sur l'augmentation des dépenses n'est pas moins important. A tranche d'âge donnée, la dépense moyenne par personne couverte est différente entre la CNSS et la CNOPS en raison de la différence entre le taux de remboursement des deux régimes, des caractéristiques liées à la population couverte comme le niveau de revenu-facteur favorisant le recours aux soins-qui est plus élevé à la CNOPS qu'à la CNSS ainsi que des habitudes de consommation en soins plus importantes chez la population de la CNOPS qui bénéficiait d'une couverture médicale avant l'entrée en vigueur de l'AMO. Toutefois, les tendances d'évolution par tranches d'âge sont presque similaires. Le test de kruskall wallis a montré que globalement l'âge est une variable explicative de la dépense médicale moyenne. Ainsi, une modification de la structure démographique de la population par âge aurait un impact sur l'évolution des dépenses.

Par conséquent, le vieillissement démographique pourrait contribuer à la hausse des dépenses médicales eu égard au fait que les personnes âgées ont des niveaux de consommation médicale plus importants. Après avoir scindé entre la population ALD et NALD, nous avons trouvé que la dépense moyenne par personne ALD n'augmente pas selon l'âge car les pathologies dont sont touchées les bénéficiaires de la couverture diffèrent d'une tranche à l'autre: les jeunes sont touchés par des maladies qui occasionnent des dépenses plus élevées. Ainsi, à la CNOPS, la dépense moyenne par personne ALD est de 18057 pour la tranche 15 - 20, 19291 pour la tranche 30 - 35 et 17879 pour la tranche 35 - 40 contre une dépense moyenne qui varie entre 13000 et 14000 pour la population ayant plus de 60ans. Pour ce qui est de la CNSS, la dépense moyenne par personne ALD est de 15280 Dhs pour la tranche 30 - 35, 17474 Dhs pour la tranche 25 - 30 et 14480 Dhs pour la tranche 35-40 contre une dépense moyenne qui varie entre 7345 Dhs et 8900 Dhs pour la population ayant plus de 60 ans.

Toutefois, le taux de prévalence en ALD croît avec l'âge et varie de 0,1% pour la tranche 0 - 1 à 19,1% pour la tranche 65-70 à la CNOPS et de 0,0% pour la tranche 0 - 1 à 19% pour la tranche 65-70 à la CNSS. Le suivi de l'évolution du taux des personnes en ALD et qui recourent aux soins a montré qu'il connaît une augmentation aussi bien pour les personnes ayant moins de 60 ans que celles ayant plus de 60 ans, ce qui veut dire que l'aggravation de la morbidité n'est pas l'apanage des tranches d'âge élevées. La dépense moyenne par personne non touchée par une ALD connaît également une augmentation selon l'âge à partir de cinq ans. Si la hausse des taux de prévalence en ALD avec l'avancement en âge explique l'augmentation des dépenses liées aux ALD chez les tranches d'âge élevées, l'évolution des dépenses non liées aux ALD selon l'âge pourrait être imputée au fait que certaines personnes touchées par d'autres maladies chroniques qui ne font pas l'objet de la liste des quarante et une affections de longue durée, ont recouru à des prestations coûteuses comme l'hospitalisation ainsi qu'à l'augmentation du taux de recours aux soins notamment les consultations, les médicaments et les examens permettant l'identification des maladies chroniques.

A la CNSS, la dépense moyenne par personne couverte non touchée par une ALD a augmenté à un taux plus élevé chez la population ayant moins de 60 ans (12,1%) par rapport à la population ayant plus de 60 ans (8,7%) durant la période 2009-2015. En ce qui concerne la CNOPS, la dépense moyenne par personne couverte non touchée par une ALD a évolué de 8,1% pour les moins de 60 ans et de 18,1% pour les plus de 60 ans. Ceci est imputé à, l'élargissement de la liste des médicaments remboursables pour les deux régimes. L'amélioration des tarifs, base de remboursement pour les consultations et visites en 2010 et des soins dentaires depuis 2014 à la CNOPS. En ce qui concerne la CNSS, l'augmentation notée est due à l'inclusion des soins ambulatoires en 2010 et des soins dentaires en 2015 dans le panier de soins qui était limité auparavant aux maladies graves ou invalidantes (soins de longue durée ou particulièrement coûteux), au suivi des enfants de moins de 12 ans, au suivi de la maternité et aux hospitalisations. Ce type de mesures engendre également l'augmentation de la part de la population qui recourt aux soins ainsi que la hausse du nombre de services utilisés par personne qui recourt habituellement aux soins. L'absence de certains outils de maîtrise des dépenses comme les protocoles thérapeutiques opposables et l'obligation du passage par un médecin traitant ainsi que l'insuffisance du contrôle médical mènent également à la dérive de la consommation médicale.

## Conclusion

L'analyse des tendances d'évolution des dépenses selon les deux groupes d'âge (moins de 60 ans et plus de 60 ans) a permis de ressortir d'autres facteurs non démographiques engendrant la hausse des dépenses médicales après un rapprochement avec les évolutions qu'a connues le contexte marocain durant la période 2012-2015. Ceci nous laisse conclure que le vieillissement démographique n'est pas le seul facteur expliquant la hausse des dépenses de santé. De plus, la contribution de ce facteur démographique ne pourrait être importante si on rapproche, d'une part, le ratio des dépenses moyennes des 60 ans et plus par rapport aux dépenses moyennes des moins de 60 ans qui n'est pas extrêmement élevé ainsi que le rythme d'évolution de la population ayant plus de 60 ans qui a progressé de 1,2 points pour la CNOPS et de 0,7 points pour la CNSS, et d'autre part, le taux d'évolution de la dépense moyenne par personne couverte beaucoup plus important. En raison de l'indisponibilité des données, nous n'avons pas pu quantifier la contribution du vieillissement démographique à la hausse des dépenses de santé et la comparer à l'impact des autres facteurs identifiés dans la littérature comme déterminants de la consommation médicale. Une étude approfondie avec des données plus détaillées nous permettrait d'étudier plus en profondeur l'impact du vieillissement démographique par le biais de simulations permettant la mesure de l'effet mécanique de l'augmentation de la part de la population ayant plus de 60 ans et d'autres tenant également compte de l'aggravation de la morbidité et du changement du profil de consommation chez les personnes âgées.

### Etat des connaissances actuelles sur le sujet

- La proximité du décès explique mieux que l'âge l'évolution des dépenses;
- D'autres facteurs non démographiques contribuent à la hausse des dépenses médicales comme l'évolution des technologies médicales.

### Contribution de notre étude à la connaissance

- Le vieillissement démographique contribue à la hausse des dépenses de santé pour les régimes d'assurance maladie obligatoire au Maroc;
- La contribution de ce facteur démographique ne pourrait être importante si on rapproche, d'une part, le ratio des dépenses moyennes des 60 ans et plus par rapport aux dépenses moyennes des moins de 60 ans qui n'est pas extrêmement élevé ainsi que le rythme d'évolution de la population ayant plus de 60 ans qui a progressé de 1,2 points pour la CNOPS et de 0,7 points pour la CNSS, et d'autre part, le taux d'évolution de la dépense moyenne par personne couverte qui est beaucoup plus important.

## Conflits d’intérêts

Les auteurs ne déclarent aucun conflit d'intérêts.
